# Implementing a Professional Development Programme (ProDeveloP) for Newly Graduated Nurses: A Study Protocol

**DOI:** 10.3390/nursrep15070243

**Published:** 2025-07-02

**Authors:** Jessica Höglander, Magdalena Lindblom, Marie-Louise Södersved Källestedt, Anna Letterstål, Margareta Asp, Margareta Widarsson

**Affiliations:** 1 School of Health, Care and Social Welfare, Mälardalen University, 721 23 Västerås, Sweden; magdalena.lindblom@mdu.se (M.L.); marie-louise.sodersved.kallestedt@regionvastmanland.se (M.-L.S.K.); anna.letterstal@mdu.se (A.L.); margareta.asp@mdu.se (M.A.); margareta.widarsson@mdu.se (M.W.); 2 Centre for Innovation, Research and Education, Region of Västmanland, 721 89 Västerås, Sweden

**Keywords:** competence, introduction programme, newly graduated nurses, professional development, recovery, role clarity, social acceptance, study protocol, task mastery, transition to practice

## Abstract

**Background/Objectives:** This study protocol outlines the implementation of a professional development programme (ProDeveloP) designed to support newly graduated nurses (NGNs). NGNs often experience inadequacy and face challenges in developing their professional competence. Healthcare organisations can ease this transition through introduction programmes. However, effective implementation strategies in local healthcare settings remain unclear. This study aims to develop, implement, and evaluate a programme that enhances role clarity, task mastery, social acceptance, stress reduction, and recovery, contributing to increased job satisfaction and retention. **Methods:** ProDeveloP will be implemented at a regional hospital in mid-Sweden. This study will include a convenience sample of 110 NGNs from both the previous and the new introduction programmes, 20 dialogue facilitators involved in the programme, and 10 managers responsible for NGNs participating in the programme. Data will be collected throughout the ProDeveloP using questionnaires, individual interviews, and focus groups. The data will be analysed using both qualitative and quantitative analyses. **Conclusions:** This study has the potential to improve NGNs’ work-related health, job satisfaction, and retention while contributing to competence development. By offering structured reflection, mentorship, and organisational support, this research project addresses knowledge gaps in understanding the long-term impacts of introduction programmes and structured reflection, contributing to evidence-based improvements in nursing practice and healthcare leadership. **Clinical trial number:** NCT06742047. Registered in ClinicalTrials.gov, 19 December 2024.

## 1. Introduction

This study protocol presents the implementation of a professional development programme (ProDeveloP), an introduction programme targeting newly graduated nurses (NGNs). During the transition from education to their new roles as registered nurses (RNs), NGNs may experience feelings of inadequacy due to limited clinical experience and encounter challenges in developing professional competence. Nevertheless, these difficulties are often accompanied by a strong willingness to learn [1]. The shift from the familiar role of a student to the less familiar role of a practising nurse can be experienced as a transition shock [2]. To develop professional competence, some prerequisites in the care culture and organisation are important for the NGNs, such as support in the care team from supervisors and colleagues and a structure within the organisation that provides opportunities for continued learning [1,3].

The development of ProDeveloP has been inspired by the report of Gustavsson and colleagues [4], who studied sustainable onboarding for new professionals, with a focus on role clarity, task mastery, social acceptance, and stress. In addition, ProDeveloP focuses on recovery as an area for NGNs’ development. The programme was developed in collaboration with Mälardalen University (MDU) and the Region of Västmanland to support NGNs during their transition into the nursing profession, helping them adapt to the highly intensive and complex hospital environment. The goal is to enhance the NGNs’ work-related health and job satisfaction and reduce the turnover rate in this group.

### 1.1. Transition from Nursing Student to Registered Nurse

The transition from nursing student to RN is a complex, dynamic, and demanding process. NGNs may experience feelings of insecurity and uncertainty, along with a need to develop an overall understanding of what it means to work as an RN [3]. This transition process begins early, upon admission to nursing education, and continues until the end of the first year as an RN. The transition can be facilitated by clinical experiences during nursing education as well as through preparation and effective routines in the workplace [5].

Colleagues and organisational support are important for the NGNs’ transition process. Research emphasises that aspects such as good working relationships and support from colleagues are central to creating a sense of belonging and facilitating the nurses’ development and performance in the clinical setting [5,6,7,8]. However, workplace interactions can also negatively influence the NGNs’ experiences, leading to feelings of isolation, struggle, or intimidation, instead of a sense of thriving and being supported, included, and validated within their profession and workplace [5].

To support NGNs in their transition from education to clinical practice, healthcare organisations can implement various onboarding initiatives, such as introduction programmes. These initiatives aim to strengthen clinical competence, build professional confidence, support integration into the workplace culture, and contribute to a more sustainable start in the nursing profession [4,9,10,11,12]. This sustainable start is important for ensuring that RNs remain in their profession. Previous research and reports have indicated that it is not uncommon for NGNs to leave the profession during their first few years [13,14,15,16]. However, transition support, such as introduction programmes, can significantly increase their commitment to both the organisation and the profession [9].

### 1.2. Being New in the Profession

The first year is described as significant for NGNs’ learning and development, a time before they have begun to feel safer and more competent [5]. Being new as a nurse often involves having certain expectations of their professional role and workplace. They frequently encounter challenges in their roles, where their expectations may not align with the realities of the work of an RN.

NGNs encounter challenges, as they are frequently expected to assume responsibilities and demonstrate competencies comparable to those of experienced RNs. For example, they may be required to prioritise patients with varying needs or assess the severity of clinical conditions, despite having limited practical experience. Furthermore, NGNs do not always work alongside experienced RNs [7]. Hence, it is not uncommon for them to experience insecurity and stress [1,8,11,17] due to their fear of being overworked or burnt out [7].

There are several reasons why NGNs leave their profession. Common factors include heavy workloads [8,18,19], inappropriate conditions, lack of knowledge or skills, communication challenges, high expectations, a negative working atmosphere and culture, ineffective support [8,20], and the challenges of transitioning from being a student [8]. Therefore, there is a clear need for support and socialisation processes for these new nurses [21].

Despite the challenges, NGNs maintain a positive attitude [8], a willingness to learn, and the motivation to develop their competence [1]. After approximately one and a half years in the profession, they describe having gained enough nursing experience to handle complex patient situations and having developed confidence and independence in their roles as RNs, contributing to their professional development [7]. Role clarity, task mastery, reduced stress, recovery, and social acceptance are all important aspects for NGNs to develop during their first year, and these factors have shown associations with lower levels of stress [4,21,22].

### 1.3. An Overview of Professional Development Programmes

Introduction programmes during the first year of practice are claimed to enhance NGNs’ readiness for healthcare practice [5]. A number of varied introduction programmes have been developed to ease the transition process for NGNs e.g., [23,24,25]. Common features in many introduction programmes are resources such as supervisors, mentorships, and peer support [23,25], with reflection and education days, but there are also many differences and a lack of consensus between current introduction programmes [23].

However, despite their differences, introduction programmes have led to several positive outcomes, such as cost benefits, high retention rates, and low turnover, when NGNs have attended programmes to ease their transition process [25]. Findings from previous introduction programmes have shown, among other things, higher levels of confidence, professional growth, and comfort among NGNs [9,26,27], with a positive impact on their sense of belonging [26] and on patient safety [9]. Laying the groundwork for a healthy transition, lifelong learning, and a sustainable working life for NGNs is important for healthcare organisations. This ensures they can attract and retain experienced and competent RNs who can function as facilitators for support and dialogue.

Previous research regarding the transition of NGNs into a new profession has shown that when NGNs experience higher levels of the socialisation process’s role clarity, task mastery, and social acceptance, they experience lower levels of stress [21]. This intensive longitudinal study prospectively followed NGNs with weekly, repeated data collections over 14 weeks. The NGNs participated in different kinds of introduction programmes in Sweden. The result indicated that to become more effective, it would be relevant to develop programmes that directly focus on the socialisation process. A preventive stress intervention was developed, and the process was evaluated to create an effective introduction [11]. The stress intervention was added to complement a traditional introduction programme and lasted for six weeks, with three sessions with face-to-face groups of 10 NGNs in each group. The result showed small statistical preventive effects on self-reported stress, and no between-group effect could be confirmed. Further research needs to be directed towards developing and evaluating more effective interventions targeting the socialisation processes. The result also showed that the transitions into the nursing profession differ between individuals [21]. Further research should consider this and conduct longitudinal studies at the individual level. There is a shortage of studies that measure outcomes at the beginning and the end of programmes [28]. Additionally, there is a lack of descriptions of how implementation strategies can successfully support transitions in local contexts. Research is also needed into balancing work-related stress with recovery and addressing the support needs experienced by NGNs. These aspects have been included in the current research project, which aims to develop, implement, and evaluate a professional development programme to support NGNs in achieving role clarity, task mastery, social acceptance, reduced stress, and recovery. This will contribute to increased job satisfaction and encourage nurses to remain in the profession.

### 1.4. Theoretical Frameworks for Becoming a Professional Nurse

To capture the transition process from nursing student to RN, this study will utilise two theoretical frameworks: Benner’s Novice to Expert model [29] and Duchscher’s Transition Stages model [2]. These frameworks provide a foundation for exploring the transition and the development of role clarity, task mastery, social acceptance, and recovery among NGNs. The following paragraphs describe these models and how they informed this study. The first theoretical model that describes the RN’s development is Patricia Benner’s comprehensive model for nursing professionalism, known as “From Novice to Expert”, which draws from the Dreyfus and Dreyfus model [29]. Benner’s model offers a valuable framework for understanding the evolution of nursing professionalism, illustrating the journey from novice to expert practitioner as a five-step progression: the novice, the advanced novice, the competent nurse, the skilled nurse, and the expert nurse, through which nurses develop and acquire professional skills. This is, however, not a linear process; RNs may progress or regress in the process, and their advancement can vary depending on the tasks and situations they encounter. Additionally, when faced with new activities or challenges, an RN may temporarily regress in their development [29].

The second theoretical framework is Duchscher’s Stages of Transition theory and Transition Shock model, which can enhance understanding of the transition process that new nurses undergo from education to professional practice, namely professional role transition [30]. The Stages of Transition theory is also described as a progressive process through three main stages: “doing” (first 3–4 months as a new RN), “being” (4–8 months), and “knowing” (8–12 months). These phases overlap with what Duchscher describes as transition shock, occurring during the first three to four months of the transition, and a transition crisis occurring around eight to nine months into the new nurses’ first 12 months of clinical practice [2].

Duchscher’s model supports Benner’s model in that NGNs in their first two to three months of practice have a more linear process of perceiving what is right and what is wrong [31]. According to Duchscher [2], during the last stage of the transition, the NGNs remain in Benner’s advanced beginner stage of clinical competence, but their focus shifts from themselves to the patient and the system they work within. They become more involved in assisting their colleagues and managing their tasks with greater experience [2].

This research project aims to develop, implement, and evaluate a professional development programme to support NGNs in achieving role clarity, task mastery, social acceptance, reduced stress, and recovery, by addressing the following research questions (RQ): 1. How do NGNs progress during the first quarter of ProDeveloP in terms of role clarity, task mastery, social integration, and stress? 2. What are NGNs’ experiences of stress, recovery, and professional development during the ProDeveloP? 3. How do NGNs perceive reflection and dialogue groups as support for their professional development? 4. To what extent are NGNs satisfied with their work, confident in their competence, and motivated to remain in their workplace after completing ProDeveloP? 5. What are dialogue facilitators and managers’ experiences of the ProDeveloP programme?

## 2. Materials and Methods

The intervention is expected to have a primary impact on role clarity, task mastery, social acceptance, stress reduction, and recovery. A secondary impact is anticipated in the form of increased job satisfaction and improved employee retention.

### 2.1. Research Design

This research project employs a quasi-experimental and exploratory design, integrating both qualitative and quantitative methods to investigate the outcomes and experiences associated with an introduction programme for NGNs, running between 2023 and 2028. It is initiated by the School of Health, Care, and Social Welfare within the Department of Caring Science at Mälardalen University, in collaboration with the Region of Västmanland and financed by AFA Insurance. Quantitative and qualitative measurements are planned, with multiple data points derived from questionnaires and interviews associated with the transition process in the early careers of NGNs during their first year in the profession.

### 2.2. Settings and Samples

Data will be collected using a convenience sample comprising NGNs, dialogue facilitators (i.e., moderators for reflection in dialogue groups), and managers. All eligible NGNs (n = 80) at a regional hospital in mid-Sweden who will participate during the first year of ProDeveloP will be asked to participate in questionnaires, focus groups, and individual interviews at the start of the programme. These participants will constitute the intervention group in this research project. Additionally, prior to the implementation of ProDeveloP, NGNs (n = 30) from a previous introduction programme will be invited to serve as a control group and will complete the same questionnaire administered to the intervention group. This questionnaire will be distributed during the third month of their programme. Furthermore, the dialogue facilitators (n = 20) involved in the programme will participate in focus group interviews, and the managers (n = 10) of the NGNs will be invited to take part in individual interviews.

### 2.3. Data Collection

**Figure 1:**
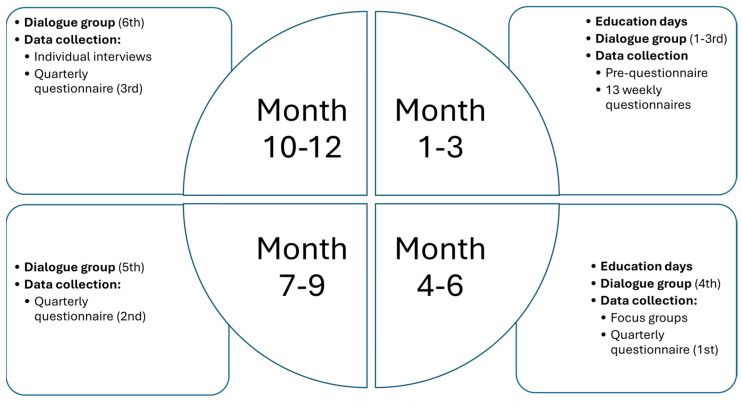
Overview of the ProDeveloP year, including data collection.

This research project will collect data using self-reported questionnaires, individual interviews, and focus group interviews. Data collection will include intensive longitudinal measures [32] consisting of both weekly and quarterly questionnaires (Table 1), as well as semi-structured interviews conducted individually and in focus groups throughout the NGNs’ first year in the profession (Figure 1).

**Questionnaires:** The questionnaires will be digitally distributed using the online survey software Survey and Report (https://www.artologik.com/se/survey-report (accessed on 6 May 2025)) [33], and will consist of validated, self-reported online questionnaires. They will be sent out throughout the year to capture the primary and secondary outcomes of the development processes and introduction support over time (Table 1). Each questionnaire will comprise approximately 40 items and is expected to take around 20 min to complete.

The questionnaires are based on a model of professional socialisation [34,35], validated in a Swedish context and used in previous studies [36,37]. Several scales of measurement will be used and distributed over time during the year of the introduction programme [4].

A pre-test will be distributed during the first week of ProDeveloP. This will be followed by intense measurements with 13 weekly questionnaires and, finally, quarterly questionnaires in months 4, 8, and 12. The week 13 questionnaire serves as the primary post-test, supplemented by the quarterly questionnaires. Two reminder notifications will be sent to participants who have not responded to the weekly questionnaires within three or five days. Each quarterly questionnaire will remain open for 30 days, with reminders sent after 5 and 10 days.

**Table 1:** Overview of the questionnaires in data collection during ProDeveloP.

Week 1	Weeks 2–14	Months 4–12
Pre-questionnaire: Demographic and contextual factors [36]. Educational factors [38,39].	Weekly questionnaires: Organisational socialisation tactics (OST) [34,40]. Agentic Engagement scale [41]. General Nordic Questionnaire for Psychological and Social Factors at Work (QPS Nordic) [42,43]. Needs Satisfaction and Frustration scale (NSFS) [44,45]. Belongingness and Uncertainty scale [46]. Copenhagen Psychosocial questionnaire (COPSOQ) [47]. Stress-Energy questionnaire (SEQ) [48,49,50,51]. Balancing Work with Recovery scale [52,53]. Emotional Demands Targeting scale [44,54]. Spare-time activities [36]. Leisure activities [36]. Karolinska Sleep questionnaire [55]. Workload scale [55].	Quarterly questionnaires: Occupational Self-efficacy scale [56]. Job Satisfaction scale [57]. Organisational Commitment on Job Turnover Intention scale [58]. Self-rated General Health Status scale [59,60]. Scale of Work Engagement and Burnout (SWEDBO) [61]. Mood and Arousal scale of emotions, depression, and anxiety [62]. Recovery, stress, and sleep items from the weekly questionnaire (see Weeks 2–14) are also included.

**Interviews:** The focus groups (n = 4) with NGNs (n = 20) will be scheduled during the intensive phase of ProDeveloP (month 4), to search for perceptions of the programme’s content and its impact on transition and professional development. The final data collection will involve individual interviews (n = 12) (month 12), focusing on experiences related to recovery, stress, support, job satisfaction, and intentions regarding leaving after the introduction year.

Focus groups (n = 5) with dialogue facilitators (n = 20) will be conducted, with questions regarding their experiences of the dialogue groups and being a dialogue facilitator. Individual interviews with managers (n = 10) will be conducted to collect their experiences of the programme, and additional questions will be asked based on the findings from the NGNs and dialogue facilitators.

All interviews will take place at the hospital and will be audio recorded and transcribed verbatim. In the focus group interviews, two researchers will participate, one acting as the moderator and the other responsible for writing memos. Individual interviews will be conducted by one researcher from the designated research group.

### 2.4. Analyses and Data Processing

Data will be analysed using both quantitative and qualitative methods. Descriptive statistics and statistical analyses will be employed to describe and compare longitudinal data, both within and between subjects, based on the questionnaire responses (e.g., regression analysis, ANOVA, Chi2) (RQ 1, 2). IBM SPSS Statistics, version 29, will be used for the statistical analyses, with methodological support and expertise provided by a statistician throughout the quantitative analysis process. Demographic information about the participants from the pre-questionnaire will be presented. The focus group interviews will be analysed using an inductive content analysis [63] (RQ 3, 5) and the individual interviews using thematic analyses [64] (RQ 4, 5).

### 2.5. Ethical Considerations

This research was approved by the Swedish Ethical Review Authority (Dnr: 2022-05682-01). ProDeveloP will be conducted at a collaborating hospital, together with the education department of that hospital. The director of health and medical care at the collaborating hospital has permitted the study. All participants will be allowed to use their work time to participate in the study. All participants, including NGNs, dialogue facilitators, and managers, will be given verbal and written information about the study, and written informed consent will be obtained by the researchers before any participation can take place. All participants will be made aware of their right to withdraw at any time, without consequences. All data will be coded and managed according to the General Data Protection Regulation [GDPR] [65]. The sampling of participants and data storage, flow, and access will be outlined following legislation and safety routines to safeguard the security, privacy, and confidentiality of participants. Data will be processed in a lawful, fair, and transparent manner. Access to participant data will be restricted to researchers involved in the ProDeveloP. All data will be securely stored on SUNET Drive, in accordance with Mälardalen University’s data protection policy. From previous studies in this context, the researchers are very experienced in the complexities and considerations needed to arrange this in an ethically sound way.

## 3. Development of the ProDeveloP

It is difficult to assess uncertainties; even with a clear theoretical base and support from existing evidence when designing an intervention, there are still no guarantees of success [66]. Hence, the design of ProDeveloP is based on previous research regarding which areas it is important to focus on to create a good introduction to the profession [1,4,6,67].

It is crucial to consider the setting where the intervention will take place and consult with various stakeholders early in the process [66]. Building strong relationships and collaborating with stakeholders involved in the development or delivery of the intervention are essential for effectively evaluating the process [68]. Therefore, the design and planning of ProDeveloP have been conducted in close collaboration with multiple stakeholders within the hospital setting where the programme will be implemented. Key collaborators and stakeholders in this programme include educator coordinators at the Clinical Skills Centre and experienced clinical nurses who will instruct and supervise in ProDeveloP, as well as the director of health and medical care, department directors, unit managers, HR strategists, and leadership developers from the Region of Västmanland. Participants in the programme, including NGNs, dialogue facilitators, clinical supervisors, and managers, will be asked to evaluate their experiences through interviews, questionnaires, and evaluation forms. This will contribute to the overall evaluation of the programme. For instance, NGNs enrolled in the programme will complete a self-assessment using a visual analogue scale ranging from 1 to 10. This assessment will measure how ProDeveloP has enhanced their sense of security in their professional role and the impact of the programme on fostering their professional growth.

### 3.1. Implementation Strategy

To guide the planning phase of the implementation of ProDeveloP, the ERIC (Expert Recommendations for Implementing Change) taxonomy [69] was used to plan and describe facilitation and to develop stakeholder interrelationships with academic partnerships. The planning of ProDeveloP was formed through collaboration in a project manager group, consisting of people from the research group at MDU, and a reference group, containing strategic people in the healthcare organisation within the Region of Västmanland. They outline the programme’s content and how it should be executed. The project manager group also supports the local expert facilitator (educational institution manager at the region) and the external facilitators (a PhD student and researchers at MDU) who are conducting and monitoring the data collection in the research. The project manager group meets annually, twice per semester, for follow-ups and re-evaluation of ProDeveloP. Such meetings are important for further development and reflections on lessons learnt [69]. In addition, an education group was put together, with representatives from the research group at MDU and representatives from the Clinical Skills Centre at the Region of Västmanland planning the educational structure of the programme. The project’s management group and the education group are key facilitators in implementing ProDeveloP at the regional hospital, while also establishing a partnership and fostering co-creation between MDU and the Region of Västmanland.

Hence, the main implementation strategy for facilitation is co-creation, which is a suitable and effective strategy for optimising the implementation of evidence into practice [70]. The implementation strategy needs an interactive approach to identify possible barriers and facilitators at various levels of the context in which the introduction programme takes place, to further support facilitation. Hence, it is important to assess facilitators and barriers in connection with development and implementation [71,72], to increase the use of evidence in practice. Facilitators and barriers occur at multiple levels, such as individual, group, unit, organisation, or in larger structural and social contexts, and there can also be interactions between various levels [71]. Some barriers, but also facilitators, identified and considered within ProDeveloP are at an individual level, such as engagement in practice change, knowledge and attitudes, and skills. Additionally, group and organisational factors, such as local routines and standards, collaboration, allocation of resources such as time and workload, and broader contexts, such as financial disincentives, must be considered in the implementation strategy. For example, time will be allocated by the clinics to allow NGNs to participate in lectures and dialogue groups during ProDeveloP.

### 3.2. Pedagogical Strategies

First, a local expert facilitator will be assigned to support two local facilitators from the clinical organisation (trained pedagogues with nursing competence and employed by the education institution), who will be responsible for the collaboration with 14 healthcare instructors (experts in their field with experience in lecturing) and two teachers for practical training (educational institution for training). The expert facilitator will also be responsible for selecting 12 dialogue facilitators (RNs who are expert and experienced in supervising), who will undergo a three-day training, which they need to acquire the required skills [73], during which they will learn about the structure and support for collegial learning in dialogue groups. Using diverse types of facilitators is also a well-known strategy for knowledge implementation in health services [74]. These dialogue facilitators are considered important to support and moderate the dialogue groups for the NGNs. Pedagogical strategies for supporting reflection in the NGNs’ dialogue groups are based on the Experiential Learning theory by Kolb. The model is ideal for learning from experiences of care and for personal and professional growth. Learning should be viewed as a continuous process [75]; therefore, NGNs need regular opportunities for reflection.

Within the ProDeveloP framework, a pedagogical strategy is to use multicomponent approaches for NGNs’ learning (Figure 1). Using such approaches has positive effects on professional practice outcomes and professional knowledge outcomes within nursing [70]. In the programme, different approaches will be used as educational strategies, e.g., face-to-face approaches (such as dialogue groups and education days with joint lectures), e-learning modalities, and reflection cards. The education days included sessions on communication, acid-base balance, patient safety, trauma and pre-hospital care, leadership, simulation/skills training, dementia, and medication management.

**Focus areas:** ProDeveloP focuses on four focus areas, all important for the socialisation process for those who are new in the workplace [35,76]: 1. Role clarity, i.e., clarity over the content and delimitation of the area of responsibility; 2. Task mastery, i.e., knowledge and skills to master the tasks that are part of the nurses’ competence; 3. Social acceptance, i.e., being part of the work team and having good relationships with colleagues; and 4. Recovery, i.e., developing strategies for rest and sleep. These four focus areas are overarching in ProDeveloP and recur during training days, during reflections in dialogue groups with dialogue facilitators and peers, and during reflection on daily work on the wards.

*Role clarity:* As an NGN transitioning into the professional realm, it is crucial to develop a clear understanding of the content and boundaries of the nursing role. Throughout the first year, reflection with colleagues is essential to gain diverse perspectives, discuss the challenges of being new, receive feedback, and contemplate one’s professional identity [4,34].

*Task mastery:* In the early stages of one’s career, new professionals must cultivate experience, knowledge, and skills relevant to nursing practice. Effective planning and prioritisation are vital. During the first year, shadowing colleagues, receiving support in planning and prioritising tasks, and reflecting on expectations and efforts are key aspects of skill development [4,34].

*Social acceptance:* New professionals need to feel included and valued within their workgroups. Actively participating in both work-related and social activities helps foster relationships. Reflecting on group expectations and teamwork dynamics with colleagues is essential for building strong professional connections [4,34].

*Recovery:* NGNs must develop a deep understanding of the importance of recovery. Reflecting on strategies for rest and rejuvenation is crucial for maintaining well-being. Viewing challenges as opportunities for growth and considering how to structure schedules for optimal recovery are important practices to adopt [4,37].

**Education:** The NGNs will participate in education days, with joint lectures, encompassing theoretical elements, skills training, and simulation activities [6] to help them prepare for different care situations that may be perceived as challenging when they are new RNs. The educational days will take place over a full day, so that NGNs can more easily attend, considering their regular work on the wards. An important strategy for planning and implementing an intervention is to take local circumstances [66] and ordinary RN work at the different clinics into account. This approach facilitates both the execution of ProDeveloP and the participation of NGNs.

A total of eight education days are included in ProDeveloP, during the first six months of the NGNs’ employment. In parallel with the programme, a specific introduction takes place on the ward where the NGNs work.

**Reflection:** To support them in their transition process, all NGNs within ProDeveloP will regularly participate in dialogue groups [77,78,79] where, together with peers and two dialogue facilitators, they will reflect upon their clinical experiences. The opportunity to reflect with peers and facilitators who are trained in conducting reflection in dialogue groups can help reduce stress among NGNs [17]. Supervision with collegial learning through dialogue groups is held six times during the first nine months of ProDeveloP.

All NGNs will also receive reflection cards showing the four focus areas (i.e., role clarity, task mastery, social acceptance and recovery), together with suggestions for reflection questions linked to each focus area. These reflection cards are designed to help NGNs explore these areas during their introduction, in dialogue groups and in their daily work on the wards.

**E-learning:** A common digital platform, administered by the Clinical Skills Centre at the Region of Västmanland, will be utilised to provide comprehensive support for NGNs, offering information about ProDeveloP, including its content, schedule, and details about the four focus areas. Additionally, participants will have access to current educational content. This platform also serves to convey further information about the research programme and its background to the participants.

## 4. Dissemination

ProDeveloP and its findings will be disseminated and communicated through web pages hosted by Mälardalen University and the Region of Västmanland, detailing progress and discoveries. Translating research findings into clinical practice remains a formidable challenge within the research continuum. We are committed to actively promoting the dissemination of findings, employing accessible language for the public and healthcare professionals, including clinicians and managers, at local and national levels. Our dissemination strategy will include presentations and seminars within organisations and professional networks. Furthermore, we will share our research findings across national and international networks, conferences, and meetings, using various platforms such as web pages and social media channels. Specifically targeting the scientific community, these findings will be disseminated to an international audience through peer-reviewed scientific publications in reputable journals.

## 5. Discussion

ProDeveloP builds on several studies aimed at developing, implementing, and evaluating a professional development programme to support NGNs in achieving role clarity, task mastery, social acceptance, reduced stress, and recovery, contributing to increased job satisfaction and the intention to remain in the profession. The content of ProDeveloP has the potential to provide evidence-based strategies for integrating NGNs into their roles as RNs during their first year in practice. Supporting these nurses during their transition from students to RNs is crucial, as this phase can often be intense and overwhelming [3,5], requiring significant support and understanding from both colleagues and managers.

There are some identified challenges with ProDeveloP, such as the need for NGNs to secure time from their managers to attend training days and dialogue groups. The introduction programme starts twice a year, meaning NGNs who join it in the autumn have often already worked during the summer, as they will have graduated at the beginning of summer. Consequently, they will have been working for a couple of months before the introduction programme starts, unlike those who begin in the spring. According to Duchscher’s Stages of Transition theory, the transition is a progressive process during NGNs’ first year in the profession, with different phases, from transition shock to transition crisis [2]. Hence, the NGNs from spring and autumn will be in different transition phases when participating in the various stages of ProDeveloP, which may affect their experiences and needs. Individual and organisational differences often pose challenges in fully adapting introduction programmes. However, this research project seeks to provide insights into how and when education days and dialogue groups can be tailored to better support NGNs in their development. Consequently, the content of ProDeveloP may need adjustments, based on findings about the optimal timing during the first year, to best meet the NGNs’ needs and developmental stages. The content of the dialogue groups, on the other hand, is well suited to adaptation based on the individual needs and conditions of the NGNs, as it is based on experiential learning to enhance their professional and personal development [75]. To participate in ProDeveloP, such as in the dialogue groups and education days, different prerequisites will be necessary, such as time and support. All NGNs will be allocated time from the clinics to participate in ProDeveloP, and managers, dialogue facilitators, educators, and peers are expected to provide support to facilitate their development and participation. The reflection cards and the digital platform will further function as facilitators for NGNs’ development in the programme.

An additional challenge is measuring the outcome of ProDeveloP, as there is a lack of validated measuring instruments for this process [28]. Nonetheless, a key strength of this study is its use of both qualitative and quantitative methods to explore NGNs’ development in ProDeveloP during their first year in the profession. By combining interviews, both individual and in focus groups, with weekly and quarterly questionnaires, the study can provide both broad and in-depth insights into NGNs’ experiences of their development within the programme. However, participation in such an intervention may influence participants’ ratings and responses, a phenomenon known as reactivity [32].

Using longitudinal measures with multiple questionnaires throughout the year is a strength, particularly the intensive measures aimed at understanding the NGNs’ individual development during the first 14 weeks of the introduction, a period often referred to as a “transition shock” for NGNs [2]. However, a challenge with intensive measurements is maintaining participant interest and minimising dropout rates, as the process can be both time-consuming and burdensome for participants [32]. To address this, it is important to follow up with weekly newsletters and reminders to help sustain participants’ commitment to completing the questionnaires. A key advantage of intensive longitudinal designs is their ability to examine relationships within and between frequently occurring behaviours, perceptions, or activities (e.g., daily, weekly), allowing researchers to explore how varying contexts or situations influence responses. Additionally, this design helps observe change processes and reduces retrospective bias [32].

Due to new directives regarding extended practice-based training in nursing education in the European Union, it cannot be ruled out that the function and content of introduction programmes will need to be revised in the future, to suit the new needs of NGNs. It is important to be sensitive to national and international changes and to create an introduction programme that best supports NGNs during their first year in the profession. Otherwise, there is a risk of difficulty in attracting and retaining nurses, leading many to leave the profession, which would negatively impact knowledge growth, continuity, and quality of care. Hence, introduction programmes such as ProDeveloP are important to enhance NGNs’ readiness for their profession [5,9,26,27] and secure sustainable work conditions and healthcare for the future. Although this research project is limited to a regional hospital, we hope the findings will provide valuable insights that can inform and enhance onboarding practices more broadly. Given the global relevance of this challenge, the results may hold significance both nationally and internationally.

The goal of improving NGNs’ work-related health, job satisfaction, and retention may be achieved through targeted studies examining the experiences of NGNs, dialogue facilitators, and managers. Empirical research on structured reflection, mentorship, and organisational support can provide valuable insights into how these processes influence professional competence, job satisfaction, and retention. Additionally, such research would help fill knowledge gaps about the long-term effects of introduction programmes and structured reflection, supporting evidence-based advancements in nursing practice and healthcare leadership. The work environment and continuous learning are crucial for newly graduated and experienced nurses. A supportive, well-structured workplace fosters professional growth and job satisfaction, which in turn improves patient care and helps maintain a healthy, sustainable work-life balance.

## Data Availability

No data was created or analysed in this study protocol. Data sharing is not applicable.

## Public Involvement Statement

No public involvement in any aspect of this research.

## Guidelines and Standards Statement

This manuscript was drafted against the SPIRIT 2013 Checklist for clinical trial protocol and related documents.

## Abbreviations

The following abbreviations are used in this manuscript:

NGN	Newly Graduated Nurse
ProDeveloP	Professional Development Programme
RN	Registered Nurse
